# Comparison of Postoperative Continuous Wireless Cardiac Rhythm Monitoring with Traditional Telemetry in Cardiac Surgery Patients: the SMART-TEL Study

**DOI:** 10.19102/icrm.2024.15085

**Published:** 2024-08-15

**Authors:** Julien Pidoux, Emilie Conus, Naomi Blackman, Javier Orrit, Gregory Khatchatourov, Patrick Ruchat, Serban Puricel, Stéphane Cook, Jean-Jacques Goy

**Affiliations:** 1 Cardiology Division, University & University Hospital Fribourg, Fribourg, Switzerland; 2 Service of Cardiac Surgery, Clinique Cecil, Lausanne, Switzerland

**Keywords:** Cardiac arrhythmia, cardiac monitoring, cardiac surgery, telemetry, wireless monitoring

## Abstract

Telemetry monitoring (conventional cardiac monitoring system [CCMS]) is a universal method for postoperative arrhythmia detection; however, the clinical challenge of alarm fatigue, primarily associated with noise or cable disconnections, persists. The introduction of wireless continuous cardiac monitoring (WCCM) represents a potential solution to enhance recording fidelity. Patients were simultaneously outfitted with both a monitoring device considered the standard of care and a novel adhesive wireless patch. A 48-h cardiac monitoring session with the two devices occurred after cardiac surgery in a unit equipped with a telemetry system. A total of 53 patients with a mean age of 60 ± 17 years were included in the trial. The number of events detected by the two systems was significantly different at 190 versus 174 for the CCMS and the WCCM system, respectively (*P* < .05). However, the percentage of agreement was not significantly different at 91% versus 88% (*P* = .37). Events were classified as follows: pause (2 events, 1%), atrial or premature ventricular contractions (18 events, 11%), atrial flutter or fibrillation (76 events, 45%), bradycardia (12 events, 7%), and tachycardia (61 events, 36%). False alarms were significantly more frequent with the CCMS (n = 21) than with the WCCM system (n = 5; *P* = .002). The study successfully demonstrated the feasibility and usability of wireless monitoring for patients requiring telemetry. The overall results are compelling, as the WCCM system performed satisfactorily, achieving results comparable to those obtained with the CCMS, even with significantly fewer false alarms.

## Introduction

Previous investigations have highlighted that routine electrocardiographic telemetry monitoring can yield false alarms, leading to mismanagement and clinical desensitization, with potentially severe consequences, including death.^1,2^ The Adverse Events in Low-risk Patients with Chest Pain Receiving Continuous Electrocardiographic Monitoring in the Emergency Department (ALARMED) study demonstrated that management was altered in just 0.2% of patients, despite an average alarm rate of 4.7 alarms per hour.^3^ This phenomenon, known as alarm fatigue, has been associated with serious cardiac adverse events, as reported in various studies.^4,5^ Notably, an observational trial revealed that only 56% of cardiac arrests in approximately 9000 telemetry-monitored patients were accurately detected.^1^

In-hospital continuous cardiac monitoring is employed for a broad range of indications. Typically occurring in the intensive care unit (ICU), cardiac telemetry involves placement of patch electrodes on the chest and a central unit worn around the neck connected to multiple cables. A wireless connection is established with a central monitoring unit at the nursing desk.

A recent qualitative study identified patient-reported issues with monitoring equipment, such as challenges during activities like showering, neck pain, and untimely alarms resulting from disconnected lead wires.^6^ Data indicate that, following premature ventricular contraction (PVC) alarms, technical alarms involving artifacts or single lead failures were the second-most frequent alarms during a 31-day ICU study.^7^ Surprisingly, the electrocardiogram (ECG) signal quality was deemed good in 73% of false alarms, raising questions about the algorithmic interpretation of such signals. Several patch-based wireless devices, including the SmartCardia (SC) device (Smartcardia, Lausanne, Switzerland), have been developed over the years **(Table 1)**, initially for ambulatory cardiac monitoring compared to standard Holter monitoring.^8^ Recently approved by the U.S. Food and Drug Administration as a patch-based adhesive recording and monitoring device with a single-lead ECG recorder, temperature sensor, and pulse oximeter, the SC device is connected to a cloud-based platform via the patient’s smartphone. An algorithm (ScaAI) offers automated real-time arrhythmia analysis. The SC device demonstrated utility in identifying a second-degree atrioventricular block as a cause of syncope.^9^ Although limited data are available comparing conventional telemetry with a wireless system, a recent trial, albeit with a small participant size and device withdrawal from the market during the study, presented inconclusive results regarding the benefits of a wireless system.^10^

This present trial seeks to assess the feasibility and usability of the SC device (Smartcardia, Lausanne, Switzerland) in postoperative cardiac surgery patients admitted to an inpatient medico-surgical unit. The primary objective is to demonstrate the feasibility of telemetry with the SC, investigate its ability to detect clinically meaningful arrhythmias, and compare results with those of conventional telemetry. Additionally, the study explores its potential in reducing false alarms, primarily attributed to telemetry interruptions, through its skin-fixation mechanism.

## Materials and methods

The local ethical committee approved this prospective study protocol (CER-VD 2020-0141), which is registered at ClinicalTrials.gov under reference no. NCT04609436.

### Study enrollment criteria

Patients aged 18–90 years who required cardiac monitoring on the inpatient medico-surgical unit at Cecil Clinic (Lausanne, Switzerland) between April 2021 and February 2023 following cardiac surgery were eligible to be included in this trial.

Separately, we excluded patients who refused to participate; patients with an allergy to the adhesive patch or a skin disease preventing the SC device from sticking to the skin; patients with an inability to follow the procedures of the study; patients already enrolled into a current study; investigators, their family members, clinic employees, and other dependent persons; patients aged <18 years; patients requiring magnetic resonance imaging examination; patients with a thoracic open wound; and patients with a severe thoracic deformity making recording not possible.

### Protocol

Written and informed consent was obtained from all patients. The participants were equipped simultaneously with a monitoring device (Philips Healthcare, Amsterdam, The Netherlands) considered the standard of care and the novel adhesive SC patch. A 48-h cardiac monitoring session using the two devices took place after cardiac surgery on the unit equipped with a telemetry system.

**Figure 1:**
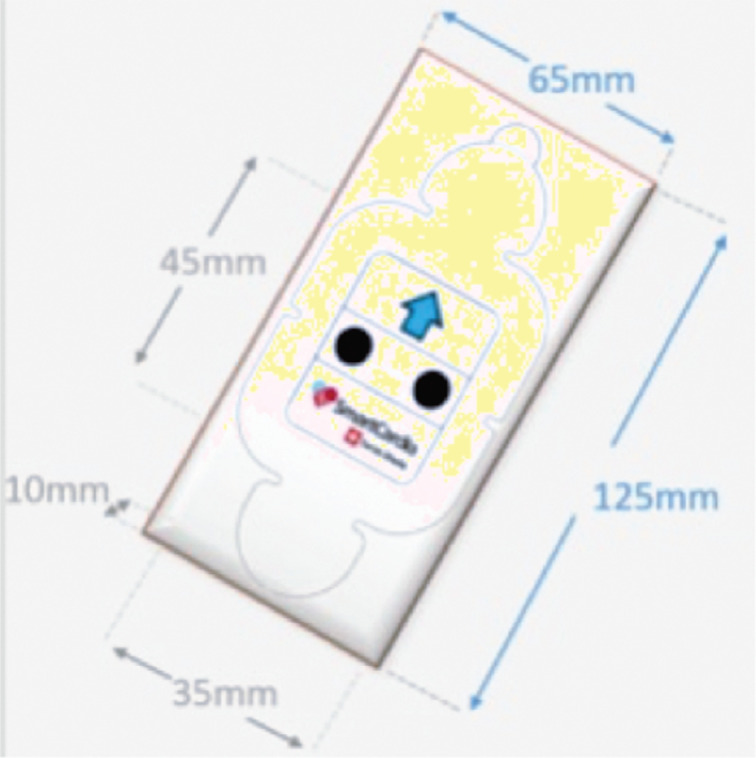
The SmartCardia device.

**Figure 2:**
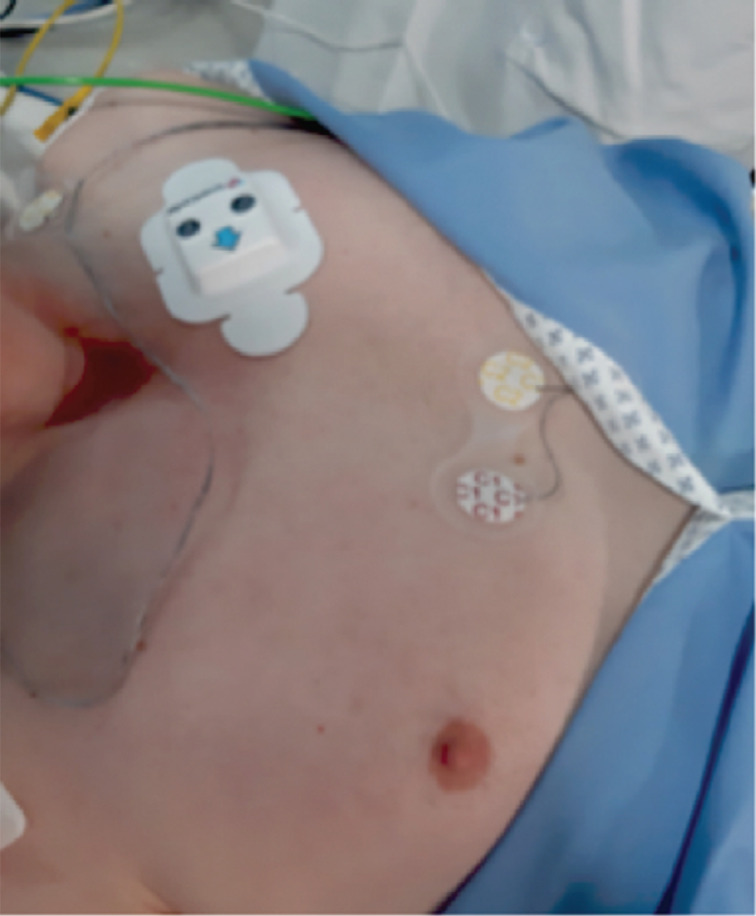
Position of the SmartCardia device on the chest.

The wireless continuous cardiac monitoring (WCCM) device used in this study, certified by both Swiss and European Union authorities, is a lightweight wireless ECG device **(Figure 1)** integrated into a waterproof patch. This entirely wireless device eliminates the need for external connection wires, allowing patients to engage in their daily activities with minimal disruption. The SC system functions as an in-hospital patient monitor, equivalent to a telemetry system, providing 24-h cardiac monitoring. Positioned on the upper left of the patient’s chest **(Figure 2)**, the device wirelessly transmits data to a screen for real-time display. Simultaneously, the data are stored in both the recording device and the cloud. Post-monitoring, the data remain accessible to physicians through the cloud. Throughout the recording, physicians can connect and query the device to visualize their patient’s arrhythmias. Initial studies employing this device in clinical settings have recently been published,^6,7^ revealing highly promising results in the ICU and daily practice. Many cardiologists have embraced the device as an alternative to the conventional Holter monitoring system.^5,10,11^

Heart rate and rhythm (sinus, pause, bradycardia and asystole, tachycardia, PVC) were compared. Standard alarms were used on the proprietary system from Philips.

Alarms triggered by each system were qualified as events. Each event was compared on both systems using the timestamp by a board-certified cardiologist. A database was completed using the population demography and the concordance and discordance of both systems, like the protocol proposed. The data from the SC system were stored on the manufacturer’s secure cloud in compliance with institutional data security policies. After the monitoring ended, the investigators reviewed the data for report generation and analysis. The report included the specifics of arrhythmia detection; in other words: sinus bradycardia <40 bpm for ≥1 min, pause >3 s, episodes of atrial fibrillation (AF), episodes of ventricular tachycardia (VT), a sustained episode of sinus or atrial tachycardia >3 min, or a premature ventricular contraction (PVC) episode lasting >3 min. The investigators compared data from the conventional telemetry flow sheet with the recordings from the SC device telemetry detections and included raw ECG waveforms of every detection.

For interpretation purposes, patients without alarms during the recording time were excluded from the analysis. Data with noise were not concerned by this exclusion.

### Analysis

The data from both systems’ telemetry were first analyzed according to their time strips, and comparisons triggered by events detected on either platform were performed. For each event, the rhythm strip was reviewed on both platforms. A certified technician was assigned the real-time SC interpretation and provided initial rhythm interpretation. Each triggered event was either assigned a rhythm interpretation or designated as uninterpretable due to unacceptable noise, as a loss of ECG signal, or designated as a false alarm. A board-certified cardiac electrophysiologist was then provided rhythm strips for final approval. Adjudicated interpretations of the conventional telemetry and SC data were subsequently evaluated for agreement.

### Statistics

Categorical variables are reported as counts and percentages, while continuous variables are reported as mean and standard deviation values or as median values with 25%–75% interquartile ranges according to their distribution. Normality was assessed by visual inspection of histograms and the computation of Q–Q plots. Given the small number of absolute frequencies for the individual detection events, *P* values were calculated for the individual detection events using Fisher’s exact test. This was an exploratory analysis not intended to reject a hypothesis. In 2 × 2 tables, the monitoring group [conventional cardiac monitoring system (CCMS) or WCCM] is represented as rows, and true-positive and false-positive results are represented as columns. All analyses were performed using Stata 18 MP 4-Core (StataCorp LLC, College Station, TX, USA).

Bar charts were created and provide a visual representation of recorded events by detailing the number of concordant and discordant events. Pie charts detailing individual event categories were likewise generated. *P* < .05 was considered statistically significant.

## Results

Fifty-three patients were monitored after cardiac surgery simultaneously via the CCMS and the WCCM system for 48 h. The demography of the studied population is shown in **Tables 1 and 2**. The WCCM system was successfully applied by the nurses in all 53 patients (100%). No adverse events, such as skin irritation, were linked to the WCCM.

**Figure 3:**
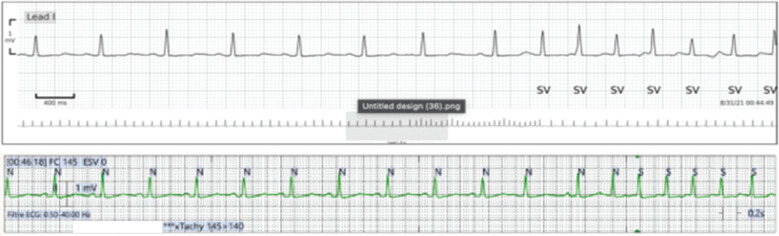
Agreement between the two systems regarding the detection of a run of supraventricular tachycardia.

**Figure 4:**
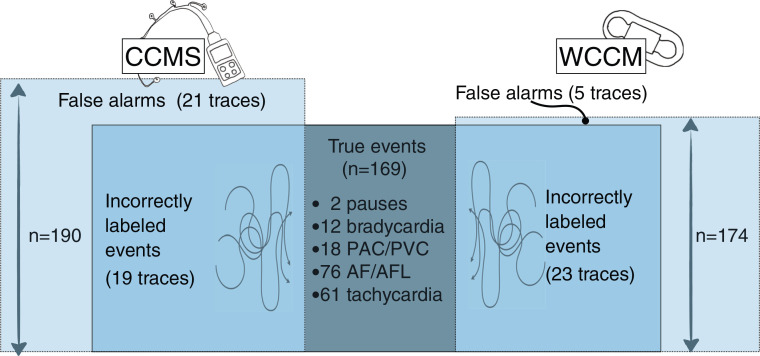
Comparison of the number of discordant traces between the two systems. *Abbreviations:* AFIB, atrial fibrillation; CCMS, conventional cardiac monitoring system; PAC, premature atrial contraction; PVC, premature ventricular contraction; WCCM, wireless continuous cardiac monitoring.

There were 190 events recorded by the CCMS and 174 events recorded by the WCCM system. Of them, 169 events were designated by the cardiologist as true events **(Table 2)**. True events were classified as follows: pause (2 events, 1%), bradycardia (12 events, 7%), atrial premature contractions or PVCs (18 events, 11%), atrial flutter or fibrillation (76 events, 45%), and sinus tachycardia (61 events, 36%) **(Figure 3)**. In the comparison between CCMS and WCCM, discordance in true alarms was noted on 26 traces (15%) **(Figure 4)**.

### Conventional cardiac monitoring system detections

**Figure 5:**
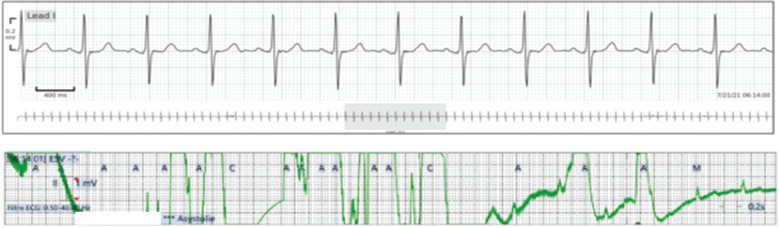
Example of a false alarm with the conventional cardiac monitoring system due to cable disconnection. The trace obtained with the wireless continuous cardiac monitoring system was good and did not show any rhythm disturbances.

**Figure 6:**
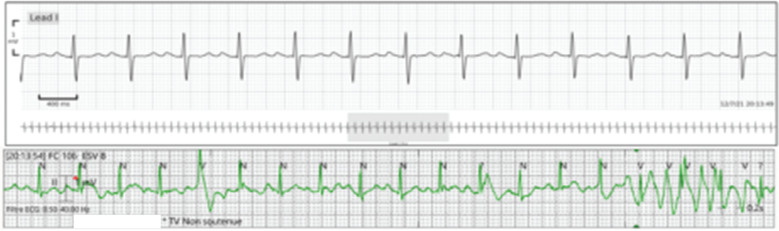
Example of a false alarm with conventional cardiac monitoring system due to movement of the patient. The artifact is falsely interpreted as non-sustained ventricular tachycardia.

**Figure 7:**
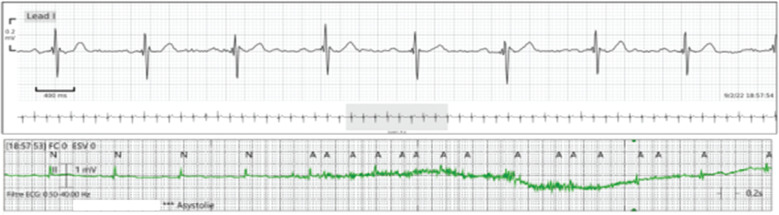
Low-quality recording obtained with the conventional cardiac monitoring system was falsely interpreted as a run of atrial tachycardia.

Among the 190 CCMS events analyzed, 21 (11%) were considered as false alarms, with most attributed to noise or cable disconnections **(Figures 4–7)**. Each false alarm corresponded to either a false pause (n = 4; 19%), false non-sustained VT (n = 7; 33%), false AF (n = 4; 19%), or false PVCs (n = 6; 29%) **(Table 3)**. Among true alarms, false event labeling was identified for 19 events.

### Wireless continuous cardiac monitoring detections

A total of 174 WCCM events were analyzed. False alarms occurred in five traces (3%). Among true alarms, false event labeling was found in 23 events, which included pause during sinus tachycardia (n = 8; 35%), non-sustained VT during sinus tachycardia (n = 7; 30%), AF during sinus bradycardia (n = 4; 17%), AF during sinus tachycardia (n = 3; 13%), and pause during sinus bradycardia (n = 1; 5%) **(Figure 4)**.

## Discussion

Telemetry is a valuable monitoring technique, facilitating continuous patient surveillance and unrestricted mobility within the hospital. However, the efficacy of this monitoring method is compromised by false alarms arising from cable disconnections. Hence, the implementation of a wireless recording system can enhance the reliability of telemetry.

This study aimed to assess the feasibility and usability of a wireless monitoring system (SC) and compare its accuracy in patients who had undergone cardiac surgery. The nursing staff effectively used the patch monitor in all cases, and no adverse side effects, such as skin allergies or device detachment, were observed.

During the 48 h of cardiac monitoring after cardiac surgery on the ward, no significant difference between the two systems was noted. We observed significantly fewer false alarms with the WCCM system, attributed to a favorable signal-to-noise ratio and the absence of cable disconnections compared to the CCMS. Prior studies comparing patch-based systems to traditional telemetry have often yielded disappointing results, with undetected events attributed to low-quality traces, noise, and ECG trace losses.^10^ False alarms pose a substantial problem in physiologic monitoring, with up to 90% of ECG arrhythmia alarms determined to be false.^7,12^ This may lead staff to lower the volume, ignore the alarm, or switch off alarms without proper patient checks, increasing risks for syncope, cardiac failure, stroke, and even death.

In our trial, the incidence of false alarms was relatively low compared to that reported in the literature,^12–14^ likely due to the nurses’ meticulous attention to electrode and device positioning and stringent alarm setting. False alarms were less frequent with the WCCM compared to the CCMS, attributed to the wireless design of the former preventing cable disconnection. Potential causes for false alarms in the CCMS include patient movement, wire entanglement, detachment during gown changes, inadvertent patient actions, and other contributing factors. Advances in technology have addressed the limitations of cable-free systems, making new wireless devices more efficient and user-friendly for both patients and staff. In a study comparing a 14-day monitoring protocol with the Zio patch (iRhythm Technologies, San Francisco, CA, USA) and conventional 24-h Holter monitoring in the outpatient setting, the patch detected more significant events.^15^ However, detecting events during the first 24 h of dual monitoring was found to be less sensitive, probably because the Holter cables were still robust. Today, cloud-based solutions like SC (ScaAi) are used in ambulatory settings as an alternative to Holter monitoring for diagnosing cardiac arrhythmias. This study showed that using SC as an in-hospital telemetry monitoring approach is feasible. SC offers an exciting alternative to standard telemetry in the ICU. The advantage of remotely viewing the traces live and permanently on any device, such as a smartphone, tablet, or computer, is also worth mentioning. This wireless system allows for immediate transmission and storage of patient data on the cloud, which is beneficial for informing doctors who are not at the patient’s bedside. The incidence of discordant traces was higher when the WCCM was defined as the reference, mainly because of false alarms or poorly recorded traces with the CCMS, due to cable disconnections.

### Limitations

Our study presents several noteworthy limitations that warrant discussion. First, the sample size represents a potential constraint, and further trials are essential to validate and confirm our findings. Second, the single-center design of this study may limit the generalizability of its results to a broader population. Additionally, each proprietary system’s definition of an arrhythmia introduces inherent limitations. While both systems used default settings, comparability was ensured by having a board-certified cardiologist review alarms. The scope of this study focused on common arrhythmias, including pause, sinus bradycardia, sinus tachycardia, AF, and VT. Further investigations are required to compare ischemic signs, such as ST-segment modifications. Finally, the storage of medical data on the cloud raises challenging privacy concerns that necessitate careful consideration and exploration in future research endeavors.

## Conclusion

The trial effectively showcased the feasibility and usability of wireless monitoring for patients in need of telemetry. The comprehensive results are compelling, as the WCCM system performed satisfactorily, achieving outcomes comparable to those obtained with the CCMS. This success can be attributed to the advancements in this new technology over the last 5 years. Notably, the safety and efficacy of the new monitoring system are on par with those of conventional monitoring systems, even demonstrating a significant reduction in false alarms.

## Figures and Tables

**Table 1:** Patients’ Baseline Characteristics

Characteristics		
Mean age (years)	60 ± 17	
	**n**	**%**
Number of patients	53	100%
Female	14	26%
Hypertension	30	57%
Diabetes	7	13%
Dyslipidemia	39	74%
Smoker	19	36%
Obesity	8	15%
CAD	34	64%
Valvular heart disease	24	45%
Peripheral artery disease	14	26%
History of arrhythmia	9	17%
COPD	2	4%
SAS	5	9%
Sinus rhythm	49	92%
LBBB	1	2%
RBBB	2	4%
First-degree AV block	4	8%
AF	4	8%

*Abbreviations:* AF, atrial fibrillation; AV, atrioventricular; CAD, coronary artery disease; COPD, chronic obstructive pulmonary disease; LBBB, left bundle branch block; RBBB, right bundle branch block; SAS, sleep apnea syndrome.

**Table 2:** Surgical Procedure

Surgical Procedure	n	%
Valvular repair or replacement	14	26%
CABG	25	47%
Vascular surgery	3	6%
Combined intervention (valvular + CABG)	10	19%
Other	1	2%

*Abbreviation:* CABG, coronary artery bypass surgery.

**Table 3:** Numbers of Total, True-positive, and False-positive Alarms

Type	Total Alarms, CCMS/WCCM	Number of Patients	True-positive Alarms, CCMS/WCCM	False-positive Alarms, CCMS/WCCM	*P* Value	Percentage of False-positive Alarms
Pause	6/2	2	2/2	4/0	0.43	67%/0
Bradycardia	12/12	5	12/12	0/0	1.00	0/0
AF, AFL	81/76	10	76/76	5/0	0.06	6%/0
Tachycardia	61/61	43	61/61	0/0	1.00	0/0

*Abbreviations:* AF, atrial fibrillation; AFL, atrial flutter; CCMS, conventional cardiac monitoring system; WCCM, wireless continuous cardiac monitoring.
